# Residual stress estimated by nanoindentation in pontics and abutments of veneered zirconia fixed dental prostheses

**DOI:** 10.1590/1678-7757-2021-0475

**Published:** 2022-04-22

**Authors:** Vinicius Pavesi Fardin, Gerson Bonfante, Paulo G. Coelho, Edmara T. P. Bergamo, Dimorvan Bordin, Malvin N. Janal, Nick Tovar, Lukasz Witek, Estevam A. Bonfante

**Affiliations:** 1 Universidade de São Paulo Faculdade de Odontologia de Bauru Departamento de Prótese e Periodontia Bauru São Paulo Brasil Universidade de São Paulo, Faculdade de Odontologia de Bauru, Departamento de Prótese e Periodontia, Bauru, São Paulo, Brasil.; 2 New York University College of Dentistry Department of Biomaterials New York United States New York University College of Dentistry, Department of Biomaterials, New York, United States.; 3 New York University Tandon School of Engineering Department of Mechanical and Aerospace Engineering Brooklyn New York United States New York University Tandon, School of Engineering, Department of Mechanical and Aerospace Engineering, Brooklyn, New York, United States.; 4 New York University Grossman School of Medicine Hansjörg Wyss Department of Plastic Surgery New York United States New York University Grossman School of Medicine, Hansjörg Wyss Department of Plastic Surgery, New York, United States.; 5 Universidade de Guarulhos Guarulhos São Paulo Brasil Universidade de Guarulhos (UNG) – UNIVERITAS, Guarulhos, São Paulo, Brasil.; 6 New York University College of Dentistry Department of Epidemiology and Health Promotion New York United States New York University College of Dentistry, Department of Epidemiology and Health Promotion, New York, United States.; 7 New York University NYU Tandon School of Engineering Department of Biomedical Engineering Brooklyn NY United States New York University, NYU Tandon School of Engineering, Department of Biomedical Engineering, Brooklyn, NY, United States.

**Keywords:** Fatigue, Ceramics, Fixed Partial Denture

## Abstract

Glass ceramics’ fractures in zirconia fixed dental prosthesis (FDP) remains a clinical challenge since it has higher fracture rates than the gold standard, metal ceramic FDP. Nanoindentation has been shown a reliable tool to determine residual stress of ceramic systems, which can ultimately correlate to failure-proneness.

**Objectives:**

To assess residual tensile stress using nanoindentation in veneered three-unit zirconia FDPs at different surfaces of pontics and abutments.

**Methodology:**

Three composite resin replicas of the maxillary first premolar and crown-prepared abutment first molar were made to obtain three-unit FDPs. The FDPs were veneered with glass ceramic containing fluorapatite crystals and resin cemented on the replicas, embedded in epoxy resin, sectioned, and polished. Each specimen was subjected to nanoindentation in the following regions of interest: 1) Mesial premolar abutment (MPMa); 2) Distal premolar abutment (DPMa); 3) Buccal premolar abutment (BPMa); 4) Lingual premolar abutment (LPMa); 5) Mesial premolar pontic (MPMp); 6) Distal premolar pontic (DPMp); 7) Buccal premolar pontic (BPMp); 8) Lingual premolar pontic (LPMp); 9) Mesial molar abutment (MMa); 10) Distal molar abutment (DMa); 11) Buccal molar abutment (BMa); and 12) Lingual molar abutment (LMa). Data were assessed using Linear Mixed Model and Least Significant Difference (95%) tests.

**Results:**

Pontics had significantly higher hardness values than premolar (p=0.001) and molar (p=0.007) abutments, suggesting lower residual stress levels. Marginal ridges yielded higher hardness values for connectors (DPMa, MMa, MPMp and DPMp) than for outer proximal surfaces of abutments (MPMa and DMa). The mesial marginal ridge of the premolar abutment (MPMa) had the lowest hardness values, suggesting higher residual stress concentration.

**Conclusions:**

Residual stress in three-unit FDPs was lower in pontics than in abutments. The outer proximal surfaces of the abutments had the highest residual stress concentration.

## Introduction

Ceramic veneer chipping and large fractures still occur in Y-TZP (Yttrium-stabilized tetragonal zirconia) systems,^1^ particularly at the marginal ridges of single crowns^2^ and in fixed dental prostheses (FDPs).^3^ A prospective clinical study reported that pontic surfaces of Y-TZP FDPs yielded higher rates of ceramic veneer fractures than abutments.^3^ This follow up study aimed to assess four- to six-unit porcelain fused to zirconia FDPs (tooth and implant-supported restorations), concluding that chipping prevalence was 20% for abutment and 80% for pontics when tooth-supported. On the other hand, with implant-supported systems, chipping prevalence was higher for abutment (55%) than for pontics (45%).

Ceramic veneer fractures have been associated with: low fracture toughness, which is characteristic of porcelain; inadequate framework support; coefficient of thermal expansion mismatch between ceramic veneer and zirconia framework;^4-8^ and subsequent veneer cooling process.^9-12^ Although residual thermal stresses are multifactorial, they are essential to the mechanical resistance of the marginal and proximal surfaces of molars,^13^ premolars,^14,15^ and connecting surfaces.^16^ Furthermore, occlusal contact location could make certain regions (*e.g.*, marginal ridges) to be more prone to fractures.^2^ Manufacturers recommend a slow cooling protocol for porcelain fused to zirconia even if all variables are controlled since the low thermal diffusivity of zirconia creates an elevated temperature gradient, especially in fast cooling protocols.^6,17^ This however does not assure a uniform distribution of residual stress among all surfaces of the ceramic veneer onto zirconia frameworks considering the presence of curved ceramic-zirconia interface, which supports a different residual stress distribution.^18,19^ Therefore, residual stress distribution in anatomically relevant specimens, including three-unit FDPs, is yet to be investigated.

Several methods for measuring residual stresses have been described for bi-layered materials. Conventional methods include Vickers hardness test,^20,21^ hole drilling,^22,23^ analytical models (such as X-ray diffraction),^10,24,25^ stress-strain analysis,^26^ finite element analysis,^6,19,27,28^ and the optical birefringence technique.^18,29^ The indentation method, particularly nanoindentation, has emerged as a powerful tool to assess the mechanical properties of materials. According to this method, low resistance caused by nanoindentation results in tensile stress (lower hardness values) whereas high resistance results in compressive stress.^30^ One main advantage of nanoindentation is that it can use the same specimen for multiple tests with a non-destructive approach.^21^ On the other hand, one main limitation is that it can only measure polished surfaces (*i.e.*, flat planes).^21^

Recent clinical data suggest that particular surfaces (*e.g.*, marginal ridges) of FDPs are more susceptible to fracture^31^ and pontics than abutment crowns.^21^ These findings have motivated the continued research efforts in the field, especially on glass ceramics fused to zirconia FDPs, which are still an unsafe surrogate for metal-ceramic prostheses.^1,32^ This study assessed residual stresses at different surfaces of a three-unit glass ceramic containing fluorapatite crystals fused to zirconia FDP, including marginal ridges and pontic surfaces, based on the hardness values obtained by nanoindentation. Two hypotheses were evaluated: (1) ceramic veneer at the pontic would result in higher residual stress than at abutment crowns, and (2) proximal marginal ridges would have higher residual stress than connector marginal ridges.

## Methodology

### Sample preparation

Maxillary teeth, including the first premolar, second premolar, and first molar, were embedded in a 25 mm diameter PVC tube with acrylic resin (Jet – Artigos Odontológicos Clássico Ltda, São Paulo, SP, Brazil). The dentin-enamel junction was positioned 3 mm above the acrylic resin. An impression was taken to reproduce the crown anatomy (Zetalabor – Zhermack, Badia Polesine, Rovigo, Italy). Then, the second premolar was removed using a cylinder diamond bur (KG Sorensen, Cotia, SP, Brazil) to create the pontic surface. The final dimensions of preparations in the first molar and first premolar abutment crowns were 2 mm of occlusal reduction, 1.5 mm of axial reduction, and a deep chamfer margin in all surfaces of the crown. An impression was taken using polyvinyl siloxane impression material (Express – 3M Oral Care, St Paul, MN, USA) to copy the prepared teeth. Abutment replicas were made by inserting composite resin (Z100 – 3M Oral Care, St Paul, MN, USA) with incremental filling (2 mm increment thickness) and light cure (Ultralux – Dabi Atlante, Ribeirão Preto, SP, Brazil).^33^ The replicas were maintained in distilled water for 30 days to allow hygroscopic expansion and to eliminate dimensional alterations after cementation of the fixed dental prostheses.^34^ Then, the composite resin abutments of the first premolar and first molar were positioned into a silicone impression created previously to embed their root portion with acrylic resin, similarly to intact teeth.

**Figure 1:**
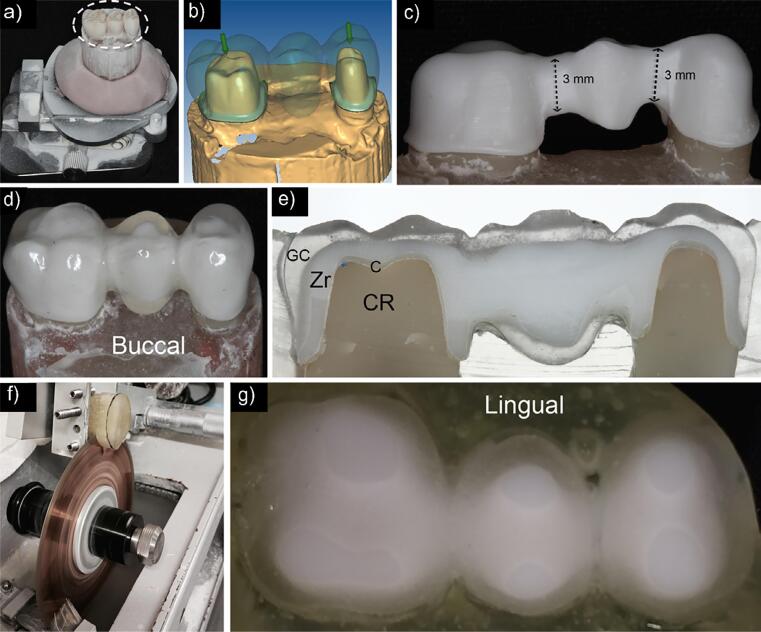
Images showing specimen preparation steps. (a) CAD/CAM spray used onto the acrylic resin replica (circle) to increase contrast before scanning. (b) The 3D FDP positioned onto the 3D abutments using the correlation option. (c) Y-TZP framework after sintering in the Sintramat furnace. (d) Hand-layered translucent glass ceramic veneer after glazing. (e) Area distribution of glass ceramic veneer (GC), zirconia (Zr), cement layer (C), and composite resin (CR). (f) Diamond saw used to apply the second cut of the FDP parallel to the metallic wire. (g) Approximate view of the specimen after cutting

A CAD-CAM system (Ceramill – Amann Girrbach, Koblach, Austria) was used to mill the Y-TZP frameworks from pre-sintered blocks (Ceramill Zl 71, 16 mm). The acrylic resin FDP replica was positioned onto the composite abutment replicas for digital scanning (Ceramill Map 400). The software created the 3D FDP image by scanning a provisional FDP made from acrylic resin (Dencôr, Artigos Odontológicos Clássico Ltda, São Paulo, SP, Brazil) and fabricated with the first impression of the unprepared teeth and the 3D prepared-abutment image of the prepared artificial teeth. Data were exported as a .stl file to use the correlation option. Abutments with framework thickness of 0.5 mm and 9 mm^2^ connector surface were created by reduction. After milling, zirconia frameworks were sintered in a furnace at 1500°C for 2 h (Figure 1 a,b,c).

**Figure 2:**
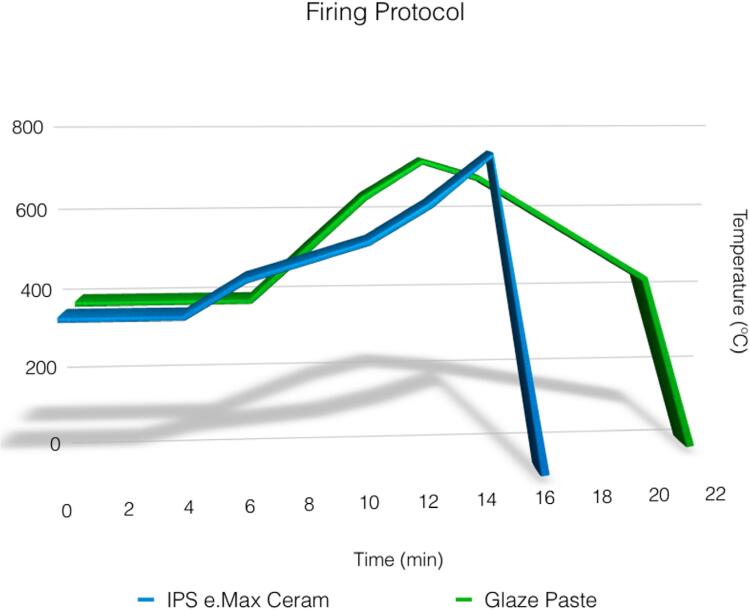
Firing protocol of ceramic veneer, IPS e.Max Ceram, and Glaze paste. The slow cooling protocol was used, following the manufacturer’s recommendations

A matrix with the anatomy of the unprepared artificial mannequin teeth was used to guide ceramic veneering. The IPS e.Max Ceram Transpa Clear (Ivoclar Vivadent AG, Schaan, Liechtenstein) hand-layered ceramic veneer, a glass ceramic containing fluorapatite crystals, was applied, resulting in 1.5 mm thickness on both axial walls and occlusal surface (Figure 1 d,e). A translucent ceramic veneer was chosen to help identify framework and veneer surfaces. The firing protocol followed the manufacturer’s recommendations (slow cooling and furnace opened at 450°C during glaze firing) (Figure 2).

Y-TZP fixed dental prostheses were cemented with a self-adhesive dual-cure resin cement (RelyX U200 – 3M Oral Care, St Paul, MN, USA) following the manufacturer’s instructions. The FDPs were maintained in distilled water at 37°C for seven days. A 0.7 mm metallic wire was customized, positioned, and fixed with acrylic-based cement (Fisher Scientific, Waltham, MA, USA) on the occlusal surface of the FDPs, following the crown’s central sulcus direction. The FDPs were then embedded in epoxy resin (Resina Epoxi RD6921, Redelease, São Paulo, Brazil) and left undisturbed until completely cured after 24 hours. Next, they were sectioned in the axial plane using a precision diamond saw (Isomet 2000 – Buehler, Lake Bluff, IL, USA) under copious irrigation. The first section was discarded whereas the thicker piece containing the FDP was cemented to an acrylic plate (Figure 1 f,g). After 24 h, the slides were ground (400 to 1200 grits Silicon carbide abrasive paper) 1.0 mm towards the cervical area of the FDPs, underwent irrigation, and were then polished (diamond suspensions of 9 to 1 µm particle size) (Buehler, Lake Bluff, IL, USA). Finally, the surfaces of each FDP were inspected using a stereomicroscope (Leica Zeiss MZE, Mannheim, Germany) to guarantee they had no surface scratches before nanoindentation testing.

### Nanoindentation testing

**Figure 3:**
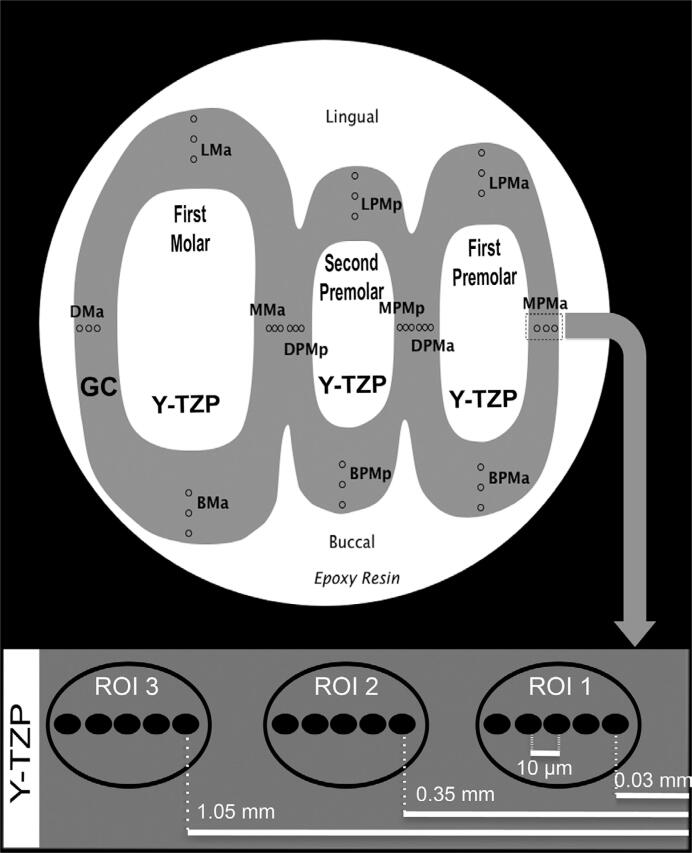
Schematic view showing group locations, zirconia framework (Y-TZP), glass ceramic veneer (GC), abutments (First Molar and First Premolar), and pontics (Second Premolar) of the specimen. The magnified view shows the nanoindentation distances of 0.03 mm, 0.35 mm, and 1.05 mm from outer porcelain surface for ROI 1, ROI 2, and ROI 3, respectively. The ROI 3 was consistently closer to the porcelain veneer/framework interface. Five nanoindentation points with 10 µm of minimum separation were performed in each spot for a more precise hardness value. The arrow shows the sequence of the nanoindentation test

The three sectioned FDPs were then assessed at the following locations: 1) Mesial premolar abutment (MPMa); 2) Distal premolar abutment (DPMa); 3) Buccal premolar abutment (BPMa); 4) Lingual premolar abutment (LPMa); 5) Mesial premolar pontic (MPMp); 6) Distal premolar pontic (DPMp); 7) Buccal premolar pontic (BPMp); 8) Lingual premolar pontic (LPMp); 9) Mesial molar abutment (MMa); 10) Distal molar abutment (DMa); 11) Buccal molar abutment (BMa); and 12) Lingual molar abutment (LMa) (Figure 3).

Nanoindentation was conducted at room temperature (23°C). A nanoindenter (TI 950 TriboIndenter, Hysitron, Minneapolis, MN, USA) equipped with Berkovich diamond three-sided pyramid was used for indentations at the ceramic veneer in mesial, distal, buccal, and lingual surfaces of the pontic and abutment crown under dry condition. The outer surface of the ceramic veneer was the reference to determine symmetric distances between three Regions of Interest (ROI) (ROI 1; ROI 2; ROI 3) in each surface of the crowns. The first indentation of ROI 1, ROI 2, and ROI 3 was conducted 0.03 mm, 0.35 mm, and 1.05 mm from the outer surface of the ceramic veneer, respectively. Each ROI received five nanoindentations with a minimum separation of 10 µm and loaded to a peak load of 4000 µN achieved in 5 seconds (800 µN/sec), followed by unloading in 5 seconds (Figure 3). Values were obtained based on description of indenter geometry by Oliver and Pharr^35^ (1992), where the following equation eliminates the plastic residual impression after unloading. The *h* is written as: *h*=*h_c_*+ h*_s_* , in which *h_c_* represents the vertical distance until contact and *h_s_* is the contact perimeter after surface displacement. This theory could also determine residual hardness. The following equations were used to obtain hardness and modulus values, beginning with:


$$E_{r}=\frac{\sqrt{\text{π}}}{2}\frac{S}{\sqrt{A}}$$

which describes the reduced modulus (*E_r_)*, the measured stiffness (*S)* and the contact area (*A)*. When the peak load is performed, the contact area created is determined by an area function F(*h*), which represents the indenter cross-sectional area to the distance from its tip, *h.* Values can be achieved by the relation: *A*=*F*(*h_c_*). The following equation should be used to obtain *F* values: *H_c_*=*h_max_*- *h_s_*. To establish hardness value, the Berkovich indenter characteristics were considered for the equations based on Oliver and Pharr^35^ (1992), resulting in:


$$H=P_{\frac{max}{A}}$$

where *H* is the hardness, *P_max_* is the maximum applied force, and *A* is the project contact.^35^ The nanoindentation software was used to generate the values automatically.

### Data analysis

The Linear Mixed Model test and Least Significant Difference test for multiple comparisons with mean and confidence intervals (95%) were conducted using the SPSS software (IBM Corp., Armonk, N.Y., USA) to compare hardness values of different surfaces of pontics and abutment crowns, especially of the marginal ridges. The tests were used to measure the differences among groups once they presented repeated values. Moreover, the rank values used are a highly efficient method to assess the distributions and find statistical difference among groups with close values. The tests are also ideal to avoid masking statistical difference between groups with significantly different values. The sum of ROI 1, ROI 2, and ROI 3 represented the total hardness of each surface of the three-unit FDPs crown. Plots were then created, in which different letters indicate statistical difference among groups.

## Results

**Figure 4:**
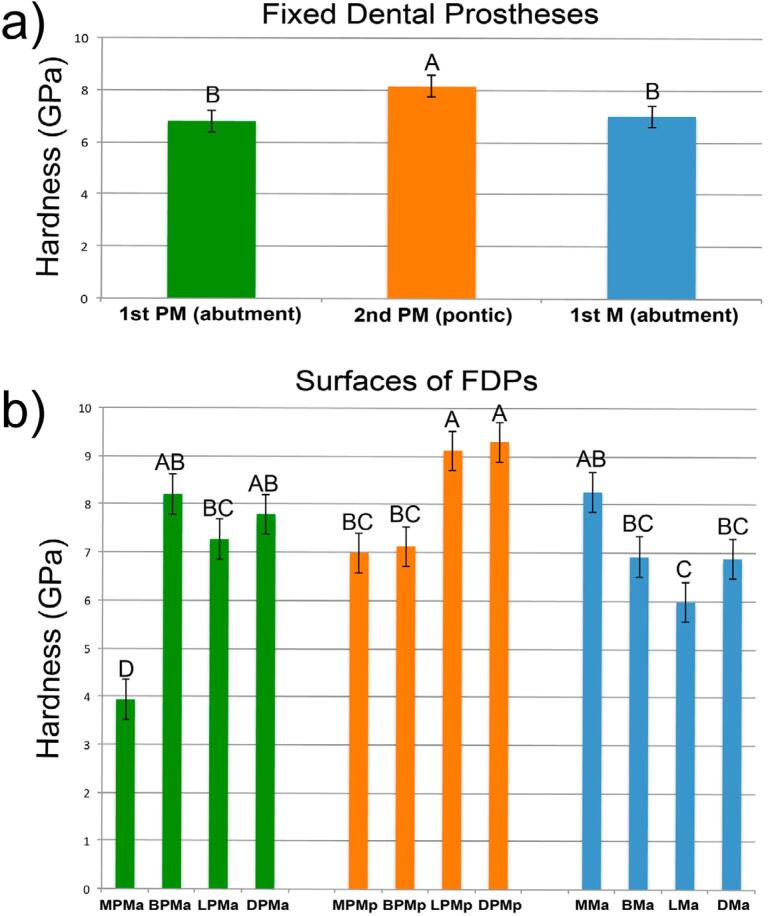
Nanoindentation results of the different ROIs of the zirconia (Y-TZP) FDP. (a) Pontic crown had the highest hardness value. (b) Proximal marginal ridges had lower hardness values than marginal ridges. Plots with different letter showed statistical difference

Overall, the sum of all surfaces (buccal, lingual, mesial, distal) of each abutment and pontic showed that ceramic veneer had the highest hardness at pontics and was statistically different than 1^st^ premolar (*p*=0.001) and 1^st^ molar abutments (*p*=0.007). No statistical difference was found between the first premolar and the first molar (*p*=0.609) (Figure 4a). Between buccal and lingual surfaces of abutments and pontic crowns, the LPMp group had the highest hardness. However, statistical differences were found between this pontic surface (higher hardness values, *i.e.*, less residual stress) and LMa (*p*<0.001), BMa (*p*=0.009), LPMa (*p*=0.028), and BPMp (*p*=0.018). The BPMa group (*p*=0.272) showed no statistical difference (Figure 4b).

A comparison between marginal ridges showed that the proximal marginal ridges of abutments (proximal abutment extremities, groups DMa and MPMa) had lower hardness values (*i.e.*, higher residual stress) than marginal ridges of connectors (DPMa; MPMp; DPMp; and MMa). Moreover, statistical differences were found between the DMa group and DPMp (*p*=0.004) and MPMa (*p*<0.001) groups and between the MPMa group and DPMa, MPMp, DPMp, MMa, DMa groups (*p*<0.001 for all groups) (Figure 4b).

## Discussion

Residual thermal stress and the low fracture toughness of ceramic veneers and masticatory force could cause early veneer failures of dental prostheses^21^. This study used nanoindentation to assess residual tensile stress in the veneer of hand-layered glass ceramic containing fluorapatite crystals fused to zirconia frameworks in fixed dental prostheses (FDPs). Data indicated that the abutment crown had higher residual tensile stress than the pontic surface, thus rejecting the first postulated hypothesis. On the other hand, a previous Vickers indentation study conducted in implant-supported FDPs showed that zirconia-veneered abutments (first premolar and molar) and pontics (second premolar) were equal when the ceramic veneer was hand-layered using a slow cooling rate for firing cycles.^21^ Besides differences in testing methodology and geometric prostheses design between the cited study (implant-supported) and the ones tested (tooth-supported), this study sought to measure residual stress 2 mm instead of 3 mm below the occlusal plane,^21^ which allowed conducting nanoindentation at connector marginal ridges. Such assessment has not been previously performed/reported and remarkably showed the lowest residual stress. A previous study by Baldassarri, et al.^21^ (2012) used two glazing layers but only a single layer was applied, which could have affected the results and thus requires further investigations. Our group had previously investigated residual stress profiles from the surface to the framework of conventional and modified zirconia framework FDPs, whose cyclic loading showed higher residual stresses than of non-loaded FDPs, increasing from the framework towards the ceramic veneer surface.^36^ Aforementioned reports and current data show that certain surfaces could have higher residual stress after processing depending on the loading area, indicating a potential early failure of ceramic veneer.

Clinical studies have reported that porcelain fractures commonly occur in surfaces of occlusal wear and surfaces without adequate porcelain support, which correlate with the surfaces of higher residual tensile stress in this study.^2,37,38^ The higher volume of the zirconia framework at the pontic could have decreased the residual stress in ceramic veneer. A previous study assessed the stress profile of the ceramic layer (1.5 mm) applied to different framework thicknesses: 0.5 mm, 0.7 mm, 1.0 mm, 1.5 mm, 2.00 mm, and 3.0 mm. The authors concluded that tensile stress was higher in the interior surface of the porcelain veneer for thinner framework.^39^

Furthermore, all-ceramic dental system processing differs considerably from industrial manufacturing of glasses and ceramics, which uses rapid cooling to increase fracture resistance,^19^ since it lacks a rigid, secondary layer material, similarly to prostheses frameworks.^19^ During the fast cooling protocol of the glaze cycle, Zirconia framework’s low thermal diffusivity (0.74 x 10^–6^ m^2^ s^–1^)^40^ increases tensile stress from the surface of the ceramic veneer towards the framework, contrary to the recommended cooling protocol for metal-ceramic restorations.^10,21,41^

The second postulated hypothesis, that proximal marginal ridges would have higher residual stress than connector marginal ridges, was accepted. This study’s results of the proximal marginal ridge corroborate with previous studies which suggested that the marginal ridges and proximal surfaces of premolar and molar crowns present the highest tensile stress.^13,14^ A photoelastic study of glass ceramic-veneered zirconia crowns showed that curved areas, including those at proximal surfaces, showed higher concentration of residual stress, independent of cooling rates.^18,19^ This corroborates with a recent experimental and finite element study^17^ which reported that curved interfaces are associated with higher stresses after evaluating the residual thermal stress of bars, semi-cylindrical shells, and arch-cubic structures bi-layered with 1.5 mm and 0.7 mm ceramic veneer and zirconia framework using slow cooling at 32°C/min and extremely-slow cooling at 2°C/min, respectively.

This study compared anatomically-relevant fixed dental prostheses with geometric shapes, including flat layers,^39,42^ bars, shell or arch-cubic structures,^17^ and virtual specimens for finite element analyses (FEA) studies.^16,43,44^ Direct comparison could have been affected by differences in geometry and methods used. Ceramic veneer distant from the framework had higher residual stress concentration and gradually decreased towards the framework, corroborating with other studies.^17,39,42^ This finding is clinically relevant since higher residual stresses primarily at the more external surfaces of the porcelain veneer could limit and amend fractures by polishing/reshaping them, thus avoiding restoration replacement. Alternatively, results of highest residual stress at proximal extremities of abutment marginal ridges corroborate with the biggest fractographic study of clinically failed all-ceramic restorations, which showed that marginal ridges seem more susceptible to failure and that occlusal contacts in this surface should be systematically eliminated.^2^

Nanoindentation was selected, thought it does not analytically measure residual stress, to estimate residual stress distribution from previous validation based on the interpretation of hardness values, where lower hardness results in tensile tress and the higher hardness results compressive stress.^30^ This methodology allowed investigating residual stress distribution 2 mm below the occlusal plane of the three-unit zirconia glass FDP surface, which included marginal ridges and pontic surfaces. This extensive analysis of residual stress distribution on FDPs surface could not be conducted by common analytical approaches, including X-ray diffraction, which is unsuitable for the glass portion of the prosthesis.^25,30^ However, further investigations are still essential to determine stress values in the different areas of FDP surface, perhaps by associating different tools.

Moreover, clinical studies have shown that connector fractures of Y-TZP frameworks are still rare, regardless of being posterior or anterior.^45,46^ Studies on the multifaceted origin of ceramic veneer residual stress and their implications on prostheses survival are therefore justified since porcelain fused to zirconia FDPs still has lower survival rates than metal ceramics.^1,32^

This study prepared composite resin teeth instead of natural teeth because of their similar elastic modulus to dentin (resin core: 16 GPa, dentin: 18 GPa),^47^ easy standardization of sample’s dimensions, and difficult access to extracted teeth. This could be considered a study limitation, though several laboratory research on dental ceramics have used composite resin substrates.^47-49^

## Conclusions

Ceramic veneer residual stress was higher at abutment crowns than at pontics in porcelain fused to zirconia three-unit fixed dental prostheses. The marginal ridges at proximal extremities of abutments showed the highest residual stresses.
